# Heterotopic Gastric Mucosa in the Gallbladder: A Rare Case and Differential Diagnosis for a Gallbladder Polyp

**DOI:** 10.7759/cureus.72372

**Published:** 2024-10-25

**Authors:** Kesley Green, Ekta Jain, Sameer Al Diffalha

**Affiliations:** 1 Pathology, The University of Alabama at Birmingham, Birmingham, USA

**Keywords:** complications, gallbladder, gastric mucosa heterotopia, heterotopia, oxyntic mucosa heterotopia

## Abstract

Heterotopia or ectopic tissue refers to the presence of normal tissue in an abnormal location, away from its typical anatomic site. While heterotopia is not uncommon, its occurrence in the gallbladder is quite rare, and even more unusual is the presence of gastric heterotopia within the gallbladder. This case report describes a 41-year-old female patient with oxyntic-type heterotopic gastric mucosa in the proximal body of the gallbladder. She initially visited the hospital for a routine follow-up for her degenerative joint disease when a gallbladder mass was incidentally discovered. Imaging revealed a circumferential, nodular wall thickening and enhancement of the proximal gallbladder body, along with mild diffusion restriction. Due to concern for primary gallbladder malignancy, a cholecystectomy was performed. Intraoperatively, the gallbladder appeared anatomically normal, with several adhesions and mild inflammation, prompting the omission of an intraoperative frozen section. Histopathologic analysis revealed oxyntic-type gastric mucosa within the gallbladder wall. Although this case of gastric heterotopia was benign, prolonged presence of gastric tissue in the gallbladder may lead to complications, such as ulceration, dysplasia, or even malignant transformation.

## Introduction

Heterotopia is defined as the misplacement of normal tissue outside of its normal anatomic location. Gastric mucosal heterotopia can be found throughout the gastrointestinal tract (GIT), from the tongue to the rectum [1]. Although heterotopia itself is relatively common, the occurrence of gastric heterotopia in the gallbladder (GB) is particularly rare [2,3]. The most common location for heterotopic gastric mucosa (HGM) in the GB is the neck, with less frequent occurrences in the body and fundus [3]. The patients are typically asymptomatic but may experience symptoms such as right upper quadrant pain, nausea, vomiting, and jaundice or present with biliary obstruction and inflammation [4-6]. HGM may appear as a pedunculated polypoid or a sessile lesion and can be seen on imaging as hyperechoic, hypoechoic, or isoechoic on ultrasound (USG) and as hyperdense or hypodense on computerized tomography (CT) scans [1,6]. To date, fewer than 100 cases of HGM in the GB have been reported. This report presents a rare case of gastric heterotopia in the GB, which, though incidentally discovered, closely resembled malignancy, prompting a thorough evaluation.

## Case presentation

A 41-year-old female patient presented to the hospital for a routine follow-up related to her degenerative joint disease. Her medical history includes hypertension, obesity, umbilical hernia, anxiety, and depression. She had previously undergone four cesarean sections, cervical and lumbar spinal surgery, and an umbilical hernia repair with mesh approximately two years before this presentation. On physical examination, her abdomen was soft, non-tender, and non-distended, with no palpable masses or hernias. The previous umbilical hernia repair incision was well healed. There was no rebound tenderness or guarding, and Murphy’s sign was negative. All other physical examination findings were unremarkable. Upon review of systems, she denied fever, chest pain, shortness of breath, fatigue, weight loss, decreased appetite, or a change in bowel movements. She did report experiencing intermittent, radiating, waist-band-like abdominal pain that resolved spontaneously without any known triggers. Additionally, she noted occasional nausea, though she had no recent episodes of emesis-her last episode of vomiting, which was non-bloody and non-bilious, occurred about a month prior to this visit. Laboratory values were all within normal reference ranges.

**Figure 1:**
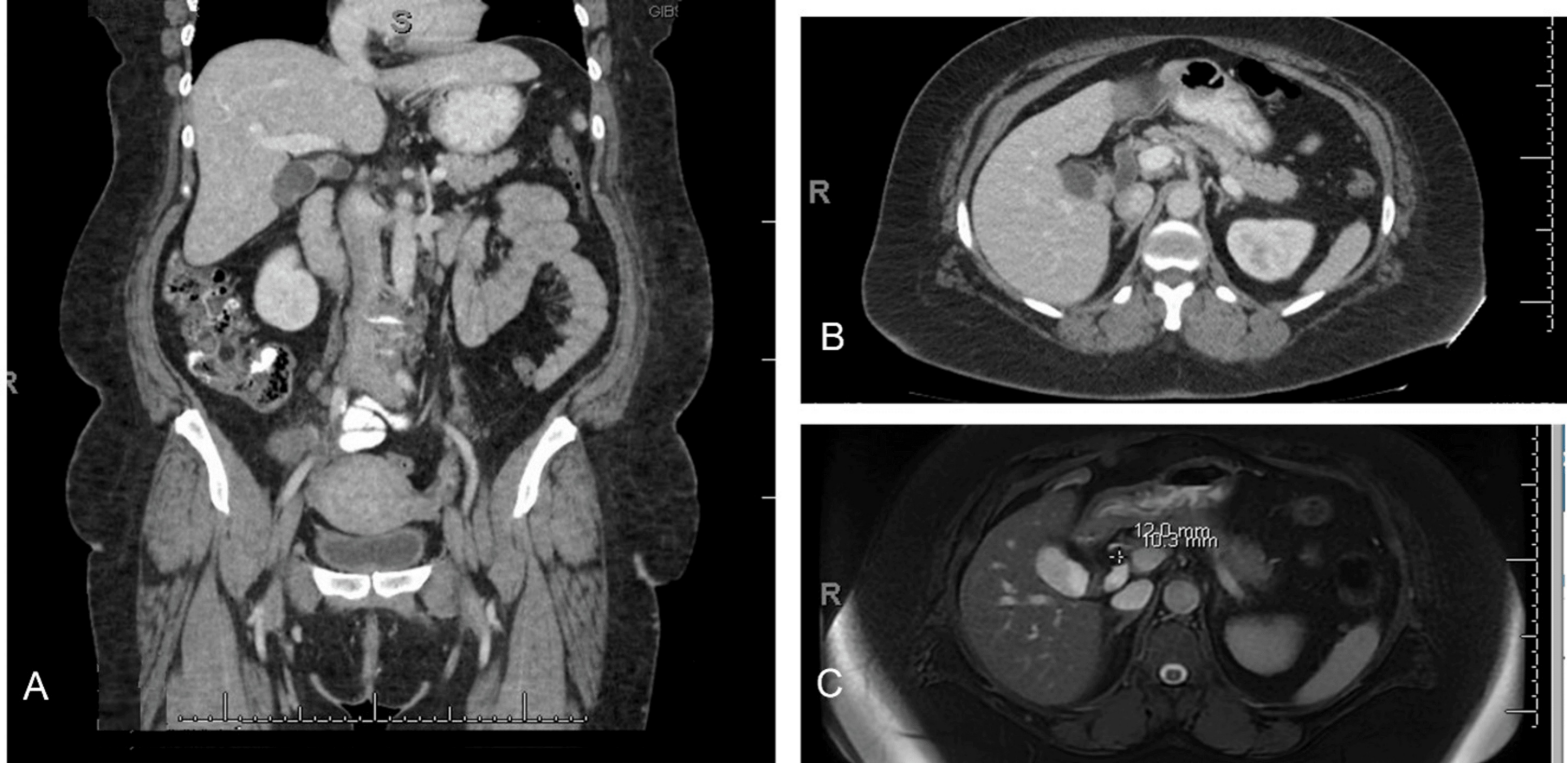
Coronal CT image (A), axial CT image (B), and axial MRI image (C) showing a circumferential, nodular wall thickening of the proximal gallbladder body with associated enhancement and mild diffusion restriction.

During a routine CT scan for her spinal condition, an enhancing mass was incidentally found at the GB neck, measuring 19 x 19 x 15 mm, along with a moderate 10-cm dilation of the GB neck. There was no intrahepatic biliary ductal dilation. Subsequent magnetic resonance imaging (MRI) and magnetic resonance cholangiopancreatography (MRCP) confirmed these findings and additionally revealed sludge and small stones. Concerned about the GB mass, she was referred to a level-one hospital for further management. The imaging there re-interpreted the mass as a 12 x 10-mm stone near the GB neck, which was likely causing a partial obstruction due to the degree of GB distension. The MRI also showed circumferential, nodular thickening of the proximal GB body wall, with associated enhancement and mild diffusion restriction, raising the suspicion of a primary GB malignancy (Figure 1). Consequently, the patient underwent a laparoscopic cholecystectomy.

**Figure 2:**
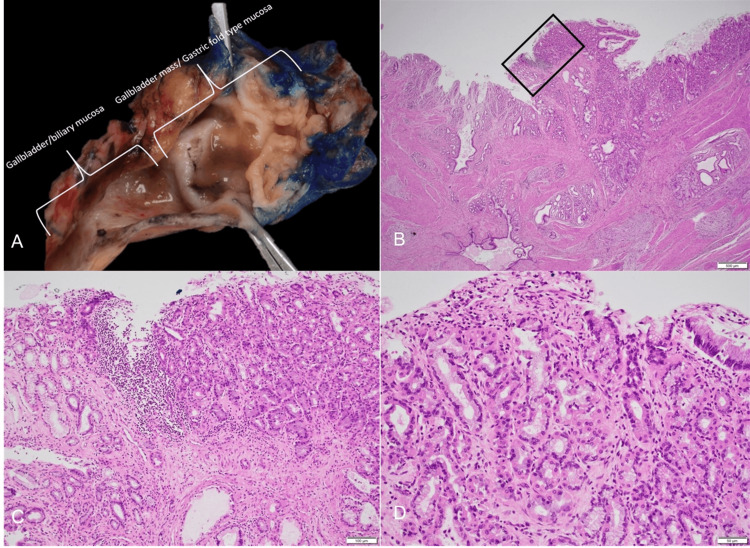
(A) Gross image of the gallbladder showing a tan-pink, circumferential, friable mass located in the gallbladder neck measuring 2.9 x 2.1 x 0.3 cm. The remainder of the gallbladder: internal surface was white-pink and trabeculated; (B), (C), and (D) show oxyntic-type mucosa with adjacent gallbladder showing chronic cholecystitis.

Intraoperatively, the GB appeared to have normal anatomy, though numerous adhesions connected it to the duodenum and omentum, indicating mild inflammation suggestive of long-standing biliary colic. A gallstone was impacted in the distal cystic duct. Given the benign appearance of the GB, an intraoperative frozen section analysis was not performed. Post-operative gross examination revealed that the GB measured 5.2 x 6.1 x 4.8 cm. The serosal surface was tan-pink, smooth, and glistening. Upon sectioning, grumous material was found within the GB neck lumen, along with a tan-pink, circumferential, friable mass in the GB neck measuring 2.9 x 2.1 x 0.3 cm. The remainder of the GB mucosa was white-pink and trabeculated. Histopathological examination showed gastric mucosa with oxyntic-type mucosa containing parietal and chief cells extending from the mucosa to the subserosa. Antral-type mucosa, Paneth cells, and goblet cells were not identified. There was associated chronic cholecystitis. The surrounding GB mucosa showed GB epithelium without significant pathological changes (Figure 2).

## Discussion

HGM is a rare condition believed to arise from three potential mechanisms: congenital anomaly during development, heterotopic differentiation, or metaplastic differentiation [1,7]. In the case presented, although congenital heterotopia is a possibility, it does not explain why it was not detected during previous imaging studies suggesting metaplasia as the underlying cause. However, lack of access to prior imaging prevents ruling out congenital HGM entirely. In our report, the patient’s age was 41 years, within the range (3-80 years) documented in the literature [6,8]. It can manifest as right upper abdominal pain, nausea, vomiting, cystic duct obstruction (with or without jaundice), and hemobilia or may be asymptomatic and discovered incidentally, as in our case [4-11]. In our study, HGM was found in the neck of GB like in previous studies where 40% of cases were found in the neck, 23% in the fundus, and 14% each in the corpus and cystic duct [6]. Our case revealed oxyntic-type mucosa, which was similar to previously reported 60% cases of body-fundic-type mucosa, 20% pyloric mucosa, and 11% antral mucosa while cardiac-type mucosa was found in none [6].

The diagnosis of HGM in GB can be difficult warranting distinction from all polypoid lesions including benign polyps, GB carcinoma, or metastasis. Radiologic imaging is usually not reliable to differentiate between benign polyps and neoplastic ones. USG is the most common diagnostic modality showing HGM to be hyperechoic rather than isoechoic or hypoechoic. Similarly, on CT, they appear as hyperdense lesions and hyperintense on MRI [6,12,13]. Additionally, Tc-99m pertechnetate scintigraphy has been used to detect HGM. This method involves injecting a radioactive substance that is absorbed by the thyroid, salivary glands, choroid plexus, and stomach and is then excreted through the urinary tract. However, this technique may be more appropriate for patients undergoing prophylactic gastrectomy due to CDH1 gene mutations, which are associated with gastric cancer, rather than for those with non-genetic HGM of the GB. If HGM is detected through Tc-99m pertechnetate scintigraphy in patients with CDH1 mutations, surgical removal of the heterotopic tissue may reduce cancer risk. In non-mutated patients, however, the risks of radiation may outweigh the benefits of the nuclear study, particularly if cholecystectomy is the recommended management regardless of the scan results [14].

Grossly, HGM appear polypoidal (sessile/pedunculated) and may develop malignant transformation (12%-52%) [6,15]. The risk of complications from HGM such as dysplasia, metaplasia, or malignant transformation is extremely low; these occurrences have been documented [16-18]. Ishikawa et al. reported that 33% of sessile GB lesions were malignant, compared to 13% of pedunculated lesions [19]. Although the size of sessile carcinomas was found to be significantly smaller than pedunculated carcinomas. Consequently, the authors concluded that all sessile lesions should undergo surgical intervention, as should pedunculated polyps larger than 10 mm due to their microscopic malignancy potential [19]. A study by Cunha et al. [20] found that an 18-mm sessile polyp adhered to the wall with central hypoechogenicity on USG, necessitating elective cholecystectomy, which on microscopy revealed gastric body and pyloric-type mucosa. Thus, a preoperative diagnosis by imaging to differentiate between carcinomatous and non-carcinomatous lesions based solely on the size and appearance of the polyp is impossible, highlighting the need for surgery. 

## Conclusions

This report highlights a rare case of oxyntic-type gastric heterotopia in the neck of the GB. HGM is a rare entity that concerns all ages and presents either asymptomatically or with abdominal discomfort, often with cholecystitis-like pain. The final diagnosis is made by histopathology. Current imaging techniques are unreliable in distinguishing neoplastic lesions from HGM, making cholecystectomy the preferred management, even when HGM is incidentally detected in asymptomatic patients. Although HGM in the GB is uncommon, heightened awareness could lead to increased identification of cases in future studies.
